# tDCS for Memory Enhancement: Analysis of the Speculative Aspects of Ethical Issues

**DOI:** 10.3389/fnhum.2016.00678

**Published:** 2017-01-11

**Authors:** Nathalie Voarino, Veljko Dubljević, Eric Racine

**Affiliations:** ^1^Institut de recherches cliniques de Montréal, Université de Montréal, McGill UniversityMontreal, QC, Canada; ^2^Bioethics Programme, Department of Social and Preventive Medicine, School of Public Health (ÉSPUM), Université de MontréalMontreal, QC, Canada; ^3^North Carolina State UniversityRaleigh, NC, USA; ^4^Neuroethics Research Unit, Institut de recherches cliniques de MontréalMontreal, QC, Canada

**Keywords:** memory enhancement, cognitive enhancement, brain stimulation, tDCS, ethics

## Abstract

Transcranial direct current stimulation (tDCS) is a promising technology to enhance cognitive and physical performance. One of the major areas of interest is the enhancement of memory function in healthy individuals. The early arrival of tDCS on the market for lifestyle uses and cognitive enhancement purposes lead to the voicing of some important ethical concerns, especially because, to date, there are no official guidelines or evaluation procedures to tackle these issues. The aim of this article is to review ethical issues related to uses of tDCS for memory enhancement found in the ethics and neuroscience literature and to evaluate how realistic and scientifically well-founded these concerns are? In order to evaluate how plausible or speculative each issue is, we applied the methodological framework described by Racine et al. (2014) for “informed and reflective” speculation in bioethics. This framework could be succinctly presented as requiring: (1) the explicit acknowledgment of factual assumptions and identification of the value attributed to them; (2) the validation of these assumptions with interdisciplinary literature; and (3) the adoption of a broad perspective to support more comprehensive reflection on normative issues. We identified four major considerations associated with the development of tDCS for memory enhancement: safety, autonomy, justice and authenticity. In order to assess the seriousness and likelihood of harm related to each of these concerns, we analyzed the assumptions underlying the ethical issues, and the level of evidence for each of them. We identified seven distinct assumptions: prevalence, social acceptance, efficacy, ideological stance (bioconservative vs. libertarian), potential for misuse, long term side effects, and the delivery of complete and clear information. We conclude that ethical discussion about memory enhancement via tDCS sometimes involves undue speculation, and closer attention to scientific and social facts would bring a more nuanced analysis. At this time, the most realistic concerns are related to safety and violation of users’ autonomy by a breach of informed consent, as potential immediate and long-term health risks to private users remain unknown or not well defined. Clear and complete information about these risks must be provided to research participants and consumers of tDCS products or related services. Broader public education initiatives and warnings would also be worthwhile to reach those who are constructing their own tDCS devices.

## Introduction

Transcranial direct current stimulation (tDCS) is a neurostimulation technique that modifies neuronal activity by delivering a weak electrical current to neural tissues through the scalp. To date, tDCS has demonstrated potential for memory improvement in both healthy people and diverse patient populations. Accordingly, tDCS is considered as a promising alternative to conventional pharmacological treatment of many diseases, including mild cognitive impairment, depression, Alzheimer’s disease, and attention deficit hyperactivity disorder—ADHD (Clark and Parasuraman, 2014). Current research is underway to determine if tDCS could reduce cognitive decline in elderly people associated with normal aging (Hsu et al., 2015). In healthy people, numerous studies show that the use of tDCS could lead to significant improvements of various cognitive functions, such as attention, language acquisition, problem solving, memory and learning, especially when coupled with training activity as learning tasks (Levasseur-Moreau et al., 2013; Coffman et al., 2014). These improvements are often described as constituting another form of cognitive enhancement.

The early arrival of tDCS on the market (Anonymous, 2013; Bikson et al., 2013; Dubljević et al., 2014) for lifestyle uses and cognitive enhancement purposes lead to the voicing of some important ethical concerns such as safety, autonomy, justice and authenticity (Hamilton et al., 2011; Farah et al., 2014). To date, there are no official guidelines or evaluation procedures to tackle these issues although current research ethics policies and laws provide a general framework. At the same time, there are several calls for the introduction of specific policies and regulatory measures, notably because of the limited knowledge of long term physiological and social implications of potential wide-spread use of tDCS (Fitz and Reiner, 2013; Maslen et al., 2014a; Dubljević, 2015). A major challenge for such policy and eventual regulation is to distinguish between substantive and imminent issues vs. trivial and farfetched ones. Accordingly, the aim of this article is to analyze the speculative aspects of specific ethical issues related to the uses of tDCS for memory enhancement. We apply the methodological framework proposed by Racine et al. (2014) for informed and reflective pro-active analysis in bioethics. This framework implies three methodological guideposts: (1) the explicit acknowledgment of factual assumptions and identification of the value attributed to them; (2) the validation of these assumptions with interdisciplinary literature; and (3) the adoption of a broad perspective to support more comprehensive reflection on normative issues.

In this light, we analyze the four major ethical considerations associated with cognitive enhancement in both conceptual and empirical literature: (1) safety; (2) autonomy; (3) justice; and (4) authenticity (Hamilton et al., 2011; Fitz et al., 2013; Farah et al., 2014). In order to assess the seriousness and likelihood of these concerns, we analyze the key assumptions underlying each of these ethical concerns based on the level of available evidence substantiating them. This analysis then allows us to reconsider such issues in light of their actual imminence and plausibility in the context of ethical analyses.

## Memory Enhancement with tDCS

tDCS devices modify neural activity by conducting weak electrical current from the entrance electrode(s) via the scalp, cranium, brain, and then back to the exit electrode(s). Depending on whether the neural tissue being stimulated is below the anodal (negative current) or cathodal (positive current) electrode(s), neuronal excitability will be respectively increased or decreased (Coffman et al., 2014). tDCS promotes neuronal activity by increasing the concentration of excitatory neurotransmitters (e.g., glutamate) and growth factors (e.g., brain-derived neurotrophic factor, BDNF) in the synaptic cleft or by acting on the membrane potential (Levasseur-Moreau et al., 2013; Coffman et al., 2014; McKendrick et al., 2015). However, the mechanisms of action of tDCS likely involve different synaptic and non-synaptic effects on neurons and non-neuronal (e.g., glial) cells and tissues within the brain (Brunoni et al., 2011). Compared to other neurostimulation devices (such as TMS or DBS), tDCS devices are relatively small and affordable (about 80 dollars when purchased online), easily portable (battery operated) and less risky (only minor side effects in healthy adults reported to date; Hildt, 2014; Pustovrh, 2014; Bikson et al., 2016). Long-lasting effects appear to modulate longer-term protein synthesis, beyond a mere short-term electronic phenomenon associated with neurotransmission. Anodal stimulation seems to increase intra-neuronal levels of calcium and neurotransmitter-receptor dependent gene expression (see Stagg and Nitsche, 2011). However, the primary mechanism of action is the polarization of resting membrane potential (see Brunoni et al., 2011), with effects lasting for up to 1 h.

The apparent effectiveness and lack of common serious side effects of tDCS, compared to pharmacological enhancers (Dubljević, 2013) and TMS (Dubljević, 2015), make tDCS a promising tool and a potential treatment for a number of diseases (Pascual-Leone et al., 2011). Indeed, recent research on different memory disorders and symptomatic memory loss identified the beneficial effects of tDCS for memory disorders such as Alzheimer’s disease (Ferrucci et al., 2008; Bystad et al., 2016), post-traumatic stress disorder (Novakovic et al., 2011), psychiatric disorders which entail working memory loss, and memory loss resulting from brain damage induced by trauma or first-stroke episode (Jo et al., 2009; Clark and Parasuraman, 2014). In healthy subjects, use of tDCS in laboratory settings seems to improve both implicit and explicit memory (Coffman et al., 2014). For example, tDCS could enhance working memory involved in motor learning as well as declarative memory when applied during specific periods of sleep (Marshall et al., 2004). Anodal stimulation with tDCS could lead to better long-term memory formation (Rroji et al., 2015), and better memory in aging, i.e., compensating for normal cognitive decline (Hsu et al., 2015; but see Fox et al., 2016). tDCS alters plasticity during learning, and perhaps also during consolidation, resulting in stronger and more persistent memories (Coffman et al., 2014).

The “easy-to-use” nature of tDCS makes it highly accessible, and tDCS devices are now available for purchase on the web for everyone interested in self-stimulation (Bikson et al., 2013). The publicly available information on the “lay” uses of tDCS even includes YouTube tutorials, which provide information for applying tDCS and for making the device from readily available components (Fitz and Reiner, 2013; Dubljević et al., 2014).

## Major Ethical Issues Associated with Memory Enhancement with tDCS

There is an emerging convergence in the neuroethics literature regarding four clusters of ethical issues associated with tDCS memory enhancement, much like enhancement uses of other biomedical technologies in healthy individuals: (1) safety; (2) autonomy; (3) justice; and (4) authenticity (Farah et al., 2004, 2014; Chatterjee, 2006; Hamilton et al., 2011; Fitz et al., 2013). These are counter-factual issues arising from legitimate or less legitimate speculations about the future, and so questions about the validity and prospectivity of these concerns remain. These questions are especially relevant for the assessment of normative and regulatory proposals. According to a preventive approach, speculation can be justified when facing scientific uncertainty in the case of reasonably foreseeable risks. At the same time, it is also necessary to engage carefully in speculation about ethical issues in order to generate a credible ethical analysis, and to assess which issues are most in need of attention. Acknowledging the prospective nature of some ethical concerns is relevant, since such focus on prospective problems may result in missing more imminent ones. However, prospective analysis in the case of tDCS is relative, as tDCS is already available on the market with the specific purpose of improving performance and, in addition, rapid advances in neuroscience suggest that limits will be surmounted in the foreseeable future (Schutter, 2014).

We first review each of these four issues as described in the literature, and then critically analyze each for its plausibility based on factual assumptions—the first pillar of informed and reflective pro-active analysis in bioethics (Racine et al., 2014). The two other steps (validation of assumptions with interdisciplinary literature, and adopting broader perspectives to support more comprehensive reflection) are carried out on the four issues collectively in the subsequent sections.

## Methodological Guidepost 1: Acknowledging Assumptions More Explicitly and Identifying the Value Attributed to Them

The four major ethical issues associated with tDCS identified above each rely in their own way on factual assumptions about the scientific aspects and societal context of the use of tDCS for enhancement purposes. Table 1 (below) identifies seven distinct assumptions: prevalence, social acceptance, efficacy, ideological stance (bioconservative vs. libertarian), potential for misuse, long term side effects, and the delivery of complete and clear information. These are regularly featured in the literature to support the existence of ethical issues associated with tDCS (e.g., Hamilton et al., 2011; Hildt, 2013, 2014; Cabrera et al., 2014; Pustovrh, 2014; Schutter, 2014). For example, the existence of coercion that would impede autonomy (one of the four major issues) is contingent on a rather strong acceptance of tDCS (an example of an assumption) and significant prevalence of the use of tDCS for memory enhancement. If tDCS was not socially accepted, coercion would be easily contested and likewise, explicit coercion would not exist unless specific environment required the use of tDCS (e.g., in legal, educational or workplace settings). Another example is the issue of safety, which will only be at stake if there are long-term side effects or if tDCS is misused by users in ways that generate additional risks.

**Table 1:** **Major ethical issues related to the use of transcranial direct current stimulation (tDCS) for memory enhancement and related assumptions**.

Ethical issues and sub-issues	Factual assumptions associated with ethical issues
Safety	− Long-term and unknown side effects − Real benefits − Potential for misuse
Autonomy
Implicit coercion	− Prevalence of use − Social acceptance
Explicit coercion	− Prevalence of use (in some specific context) − Social acceptance − Efficacy
Breach of informed consent	− Access to clear and complete information about risks and benefits
Justice
Distributive justice	− Efficacy − Prevalence of use
Fairness	− Efficacy − Prevalence of use − Ideological stance
Authenticity
Individual authenticity	− Efficacy − Ideological stance
Collective authenticity	− Efficacy − Ideological stance − Prevalence of use

Our critical reflection relies on an analysis of the current state of knowledge about each of these seven assumptions in order to determine their plausibility. By analyzing current knowledge, we show that the four ethical issues identified above involve factual assumptions. The value of such assumptions in prospective ethical analysis is sometimes acknowledged explicitly by authors (e.g., Lapenta et al., 2014; Farah, 2015) but often it is not. In order to identify how realistic and scientifically well founded these assumptions are, we classify ethical concerns and risks based on the plausibility of the assumptions they rely on.

### Issue 1. Safety

#### Identification of the Underlying Assumptions

One of the first ethical preoccupations linked to the use of tDCS for enhancement purposes relates to safety (Cabrera et al., 2014; Hildt, 2014; Pustovrh, 2014). As tDCS is considered non-invasive and used in research settings on numerous people with only benign and transitory side effects, this device is usually considered to be relatively safe (Bikson et al., 2016). However, in the ethics literature, authors worry about the potentially dangerous long-term and unexpected side effects of tDCS use, leading to some safety concerns (assumption about long-term and unknown side effects). In the evaluation of a device like tDCS, the actual benefits (assumption about real benefits) come into play since they are balanced with possible harms. Notably, harms of tDCS also include the enduring of side-effects associated with potential misuse, overuse, or abuse (assumption about the potential for misuse).

#### Explanation of the Role of the Assumptions

##### Assumptions about long-term and unknown side effects

The risks associated with tDCS, such as headaches, burning sensations and fatigue (Poreisz et al., 2007) are those that are actually occurring in the research setting and being reported in the scientific literature. However, additional unexpected risks could appear in cases of use outside of controlled laboratory settings, especially considering the potential repetitive and long-term use by healthy subjects facilitated by the online availability of tDCS (Hildt, 2014; Pustovrh, 2014) and risks associated with “transfer” of this technology to pediatric and other vulnerable populations (see e.g., Davis, 2014). Concerns about safety are related to the lack of knowledge and scientific uncertainty regarding unexpected or long-term side effects of tDCS enhancement uses (Hildt, 2013; Lapenta et al., 2014). Thus, these concerns are linked to the probability of long-term side effects or unexpected side effects, as short-term side effects reported to date are generally benign and transitory.

##### Assumptions about real benefits

The evaluation of side effect needs to take into account the efficacy and real benefits of enhancement with tDCS. Indeed, the level of reasonably acceptable risk depends on the level of reasonably expected benefits.

##### Assumptions about the potential for misuse

Safety concerns suppose that some users will “misuse” the device (see e.g., Dubljević, 2015). This is especially true for ethical considerations related to websites selling tDCS devices and YouTube tutorials providing information for the assembly and use of homemade devices. As there are no official guidelines for the use of neuroenhancement, some authors worry about the appropriate intensity, stimulation duration, exact placement of electrodes on the scalp and frequency of use (Fitz and Reiner, 2013; Dubljević, 2015).

Thus, safety concerns are based on the assumptions that tDCS could be dangerous if misused or because of long-term side effects, and that this risk will outweigh benefits (real benefits of tDCS in a benefits-risks ratio assessment).

### Issue 2: Autonomy

#### Identification of the Underlying Assumptions

Concerns about memory enhancement involve a potential challenge to individual autonomy, notably through different types of coercion (implicit: by social pressure, or explicit: by requirements from some authority) or through the breach of informed consent (Farah et al., 2004; Chatterjee, 2006; Cabrera et al., 2014; Lapenta et al., 2014). A breach of autonomy by social pressure (implicit coercion) resulting from the productivity and excellence imperatives of Western societies is worrisome (CCNE, 2014; Cabrera et al., 2014; Farah et al., 2014). This scenario relies on tDCS use becoming common (assumption about prevalence of use). If performance pressures mount, one might question the liberty of choice to use electrical neurostimulation to improve cognitive performance and memory. This coercion could become explicit if enhancement was practiced or required by parents (for their children), military forces (for soldiers), or employers (for their employees; Farah et al., 2004; Hamilton et al., 2011; Cabrera et al., 2014; Maslen et al., 2014b). Such social influence and acceptance (assumption about social acceptance), and especially in the case of explicit coercion, could potentially undermine individual freedom to choose to enhance or not (Schutter, 2014). A potential violation of an individual’s informed consent is also worrisome for those who might have to use these devices, given the lack of knowledge regarding risks and benefits, as described above (assumption about access to clear and complete information about risks and benefits; Hamilton et al., 2011; Cabrera et al., 2014; Hildt, 2014).

#### Explanation of the Role of the Assumptions

##### Assumptions about prevalence of use and social acceptance (implicit coercion)

The emergence of implicit coercion through social pressure necessarily requires that some individuals or groups perceive a significant prevalence of use. Indeed, we should worry about pressure exerted on individuals both in specific contexts (e.g., in academic or work settings) or in society at large only if a majority, or a minority of those that are highly successful, actually enhance a given function (e.g., memory). If no one is enhancing cognitive functions with tDCS, there would be no pressure for others to enhance to the level of tDCS users. In addition, if a majority of people use tDCS for enhancement (e.g., for improving memory), this is necessarily linked to the presence of a high level of tDCS enhancement acceptance. A minimally accepted technology will not be used as much. Implicit coercion is not necessarily linked to the efficacy and safety of the use of tDCS, provided that side effects do not become catastrophic, where a halt in use could be assumed. In sum, the emergence of implicit coercion is based on two assumptions: a certain threshold of prevalence and broad public acceptance of the device.

##### Assumptions about prevalence of use, social acceptance and efficacy (explicit coercion)

The concern that there will be explicit coercion presupposes the emergence of a requirement of tDCS use in specific contexts, such as legal, educational, military or workplace settings. Competitive spheres where memory enhancement is especially relevant could easily be affected—for example, as a means to perform better on an exam. In addition, efficacy is implied by the potential emergence of such requirements, as well as at least a minimal guarantee of safety parameters. Indeed, it is difficult to imagine the emergence of such requirements if tDCS was known to be dangerous and non-efficient. Thus, we find that there are three necessary assumptions underlying the appearance of explicit coercion: prevalence of use (especially in some specific context), social acceptance and efficacy of tDCS in real life settings.

##### Assumptions about access to clear and complete information about risks and benefits

Finally, concerns about breaches of autonomy and informed consent imply that clear and complete information is not provided to users. Some individuals might naively overestimate benefits and underestimate risks, an especially plausible scenario because tDCS is widely considered to be non-invasive, potentially leading to an unduly positive perception of safety (Davis and van Koningsbruggen, 2013; Fitz and Reiner, 2014; Hildt, 2014). Furthermore, many authors caution that promising enhancement of cognitive performance and memory is not advisable at this time, since more research is necessary to validate potential benefits (Hildt, 2013; Clark and Parasuraman, 2014; Coffman et al., 2014; Duecker et al., 2014; Pustovrh, 2014). There are worries that an accurate, evidence-based risk-benefit profile is difficult to obtain at this time, and that this leads to a breach of users’ informed consent (Hamilton et al., 2011; Cabrera et al., 2014; Hildt, 2014). Additionally, given that external stimulation such as tDCS could modify physiological parameters that play a role in individual behavior, some express additional concerns about a risk to the provision of *valid* consent (Cabrera et al., 2014). This concern is potentially relevant in the case of clinical uses of tDCS on people with psychological and neurological disorders (who are more likely to have difficulty giving valid consent), but less relevant in the case of use by healthy people. Accordingly, a breach of informed consent is based on the assumption that tDCS users will not have access to complete and clear information about the real risks and benefits of tDCS.

### Issue 3: Justice

#### Identification of the Underlying Assumptions

Widespread use of tDCS (assumption about prevalence of use of tDCS for memory enhancement and assumption about efficacy) may represent a challenge to social and distributive justice, due to the fear of the emergence of a class of “neuroenhanced” persons (Farah et al., 2004; Hamilton et al., 2011; CCNE, 2014). As neuroenhancers such as tDCS are being developed in greater numbers, there are concerns about their equitable distribution, which could be hampered by financial and social barriers, potentially reinforcing the gap between people with low vs. high socio-economic status, particularly in the contexts of education and employment (Farah et al., 2004; Hamilton et al., 2011; CCNE, 2014). Using tDCS may also challenge fairness, especially in competitive settings. These meritocratic concerns refer to a specific vision of work and effort: “no pain no gain” (Chatterjee, 2006). Accordingly, increased performance using tDCS could be viewed as “cheating” (Madan, 2014). There are two aspects of tDCS use that could contribute to the view that enhancement uses would in fact be cheating: breaking formal or informal rules or seeking an unfair positional advantage in competitive settings. The salience of these concerns hinges on the underlying biolibertarian or bioconservative stance (assumption about ideological stance).

#### Explanation of the Role of the Assumptions

##### Assumptions about efficacy and prevalence of use (distributive justice)

Concerns related to distributive justice are based on the assumption that tDCS will not be equally distributed. Such concerns seem clearly plausible in Western societies^1^ since there already are a large number of inequalities (Farah et al., 2004; Chatterjee, 2006; Hamilton et al., 2011). Also worrying is the potential solicitation of health professionals in the use of these technologies for treating cognitive disorders which would be part of the spectrum of “normal”, what some call the “slippery slope” of the clinical need (Cabrera et al., 2014). This solicitation raises other issues relating to the allocation of health resources and the nature of clinical duty and expertise regarding the distribution of biomedical devices with enhancement aims (Forlini et al., 2013; De Ridder et al., 2014; King et al., 2014). Furthermore, there are concerns that stimulation for personal and lifestyle use by healthy people will not be supported by medical insurance, similarly to psychostimulants (Hamilton et al., 2011), restricting use to people who can pay out of pocket for such improvement. Non-enhanced individuals could then potentially be seen as individuals with pathological brain states (Hamilton et al., 2011). That said, justice issues related to tDCS are relevant only in the case of an effective technology, leading to proven benefits and real improvements of cognitive performance or memory functions. Indeed, there are no real concerns regarding a fair distribution of a device that does not work or does not bring real benefits to users. Moreover, because a natural inequality already places people at different levels of cognitive performance, some have argued that neuroenhancement could reduce these inequalities (Greely et al., 2008). In this sense, the principle of justice may be invoked to defend enhancement uses of tDCS in order to overcome this natural gap improving individuals with deficits not connected to a specific pathology or simply those who are less efficient (Greely et al., 2008). From this perspective, accessibility of tDCS technology for enhancement would even constitute a moral duty. However, whichever stance one takes in terms of justice and enhancement uses, the normative conclusion can only hold in the case that tDCS is in fact efficacious. Concerns about justice are also related to prevalence: enhancement use of tDCS is unfair only if some users have access to this device and others do not, and if there are formal or informal rules regarding such use. Thus, two assumptions underlie justice concerns: actual efficacy of tDCS and prevalence of use.

##### Assumptions about ideological stance and assumptions about efficacy and prevalence of use (fairness)

In addition to assumptions about efficacy and prevalence of use, concerns about fairness are linked to a certain ideological stance, notably in the context of the debate between biolibertarians and bioconservatives. Namely, it would be justified to enhance cognitive function (from a biolibertarian perspective) or not (from a bioconservative perspective). Indeed, the development of tDCS uses within competitive environments relies on meritocratic arguments, comparable to arguments about sports doping (Nielsen and Cohen, 2008). Questions concerning enhancement legitimacy do not spare the use of tDCS for memory enhancement (Nielsen and Cohen, 2008; Hamilton et al., 2011; Pustovrh, 2014). These concerns are real in environments where it could be considered that if these improvements are neither authentic nor deserved, then they are not morally commendable (Caplan, 2004; Hamilton et al., 2011). Thus, assumptions about the actual efficacy of tDCS, prevalence of use and ideological stance underlie fairness concerns.

### Issue 4: Authenticity

#### Identification of the Underlying Assumptions

Finally, the development of enhancement technologies poses generic concerns about authenticity and personality. If tDCS devices actually modify memories (assumption about efficacy), this could be seen as a possible threat to self-identity and the meaning of our lives (Farah et al., 2004; Chatterjee, 2006; Racine, 2010; CCNE, 2014), notably the meaning and nature of human excellence and happiness (Racine, 2010). At the extreme, questions about authenticity may concern the modification of “human nature”—related to identity and personality in particular (Hamilton et al., 2011; Cabrera et al., 2014). This concern is relevant at both the individual (changing personality) and collective levels (changing the nature of the human species). Depending on the adopted worldview (biolibertarian or bioconservative) these changes will be seen as positive or negative (assumption about ideological stance).

#### Explanation of the Role of the Assumptions

##### Assumptions about efficacy and assumption about ideological stance (individual authenticity)

It is not clear whether it is probable that tDCS used to improve cognitive performance can affect collective authenticity. This question seems reasonable if one is interested in improving mood or social cognitive characteristics as patience or empathy (Hamilton et al., 2011). However, these improvements are highly dependent on the actual efficacy of the device. Major changes to our species (collective authenticity) or to each personality (individual authenticity) would require a substantial change for both working memory, for which there is some evidence, and long-term memory for a substantial portion of the population. In addition, it should be noted that the considerations relating to authenticity greatly depend on ideological stance. Indeed, whether such modifications could be seen as risky for authenticity or as benefiting any single human crucially depends on whether one adopts a bioconservative or biolibertarian worldview. Response to these concerns is thus linked to two assumptions: efficacy and ideological stance.

##### Assumptions about efficacy, ideological stance and prevalence of use (collective authenticity)

Human species modification on a great number of individuals also presupposes a high prevalence. Thus, a breach in collective authenticity strongly depends on efficacy, ideological stance and prevalence of the device use.

## Methodological Guidepost 2: Comparing Expectations (Assumptions) with Other Literature

Acknowledging the existence of assumptions is a first step, but the critical analysis of these assumptions needs to be carried out to assess the plausibility of individual ethical issues. In order to achieve this, we scrutinize the assumptions identified above in light of the available empirical data and scientific literature.

### Assumptions About Long-Term and Unknown Side Effects

Several non-exclusive conceptual models have been offered in order to explain the underlying mechanisms of tDCS effects on the brain (Levasseur-Moreau et al., 2013). One of them is a “zero-sum theory”, also known as “addition-by-subtraction” (Levasseur-Moreau et al., 2013; Brem et al., 2014; Luber, 2014). This model assumes that neural capacity is limited, meaning that the brain has a determined total capacity, which implies that a cognitive gain is necessarily accompanied by a negative impact on one or more other functions (Levasseur-Moreau et al., 2013; Brem et al., 2014). Accordingly, the enhancement of performance would be the result of a reallocation of brain resources (Levasseur-Moreau et al., 2013; Brem et al., 2014). Brain mechanisms responsible for the desired performance would be privileged, to the detriment of competing neural networks responsible for non-essential or less essential needs. The validation of this model is necessary because it would bring more attention to the undesirable side effects of neuroenhancement, which have been poorly investigated to date (Luber, 2014). Indeed, there is some evidence that electrical stimulation could enhance some cognitive functions only to the detriment of others (Iuculano and Cohen Kadosh, 2013; Brem et al., 2014; Kadosh, 2015). Notably, Iuculano and Cohen Kadosh (2013), in one of the first studies investigating dual-dissociation tasks with transcranial neurostimulation technologies, demonstrated a cognitive cost associated to numerical learning tasks. Application of transcranial electrical stimulation lead either to an improvement of the learning rate but not of the automation process, or to the exact opposite, depending on whether one stimulates the posterior parietal cortex or the dorsolateral prefrontal cortex, respectively. This cognitive cost could mainly be due to a change in the metabolic consumption and neurochemical modulation after electrical stimulation (Iuculano and Cohen Kadosh, 2013). However, the “zero-sum theory” model is only appropriate when stimulation induces changes in a limited capacity system and does not apply to a situation where non-invasive brain stimulation (such as tDCS) increases overall resources (Luber, 2014). Thus, in many cases, identifying a cost is difficult. For certain functions, it may be that the brain is not finite and can always enhance its capabilities, and sometimes stimulation may add to the resources available (Luber, 2014).

For now, side effects of tDCS are minor and transitory, even if some exceptional cases of syncope (black-out), seizure or transient respiratory arrest have been reported (Nitsche et al., 2003; Poreisz et al., 2007; Pustovrh, 2014; Dubljević, 2015; Ekici, 2015). tDCS could also induce phosphenes (subjective perception of short light flashes) if the current is suddenly removed (Nitsche et al., 2003). Since the underlying brain mechanisms of this technology are little understood at this time, the cognitive effects of tDCS neuroenhancement deserve further investigation and attention. More studies should be performed in order to validate the designation of tDCS as safe outside the laboratory setting. Long-term side effects have not been sufficiently investigated to support a move to daily use, and no formal or official guidelines are currently prescribed for potential private use (Fitz and Reiner, 2013; De Ridder et al., 2014; Hildt, 2014). To date, there is no evidence for irreversible injury produced by conventional tDCS research protocols and no serious adverse side effects in trained tDCS use, as reported by Bikson et al. (2016) in a review of studies grouping 33,000 sessions and over 1000 subjects who received repeated tDCS sessions. However, several notes of caution have been raised for self-directed stimulation including potential overuse, unexpected effects with inappropriate dose and the long term use, as well as variable naturalistic setting of home-use (Bikson et al., 2016).

tDCS could also interact with other existing medications; this has been scarcely investigated so far, along with the potential effects of other factors such as gender, age and handedness (De Ridder et al., 2014; Fitz and Reiner, 2014). It is possible that certain existing medications could enhance tDCS effects. Also, neuronal excitability, on which tDCS has an effect, is by nature very variable between one individual to another (De Ridder et al., 2014; Horvath et al., 2014). Therefore, individual differences related to plasticity and tolerance should be explored in-depth, especially since there is some evidence of addictive reactions to electrical stimulation in animals (Heinz et al., 2012, in Levasseur-Moreau et al., 2013). Moreover, some authors acknowledge the need for more studies investigating the effects of tDCS on pregnant women and children (Minhas et al., 2012; Maslen et al., 2014b). This is problematic because there are currently no restrictions on who can use tDCS outside of clinical and research setting, and a recent case of seizure induced by tDCS in a pediatric patient has been reported (Ekici, 2015). Short-term changes in brain plasticity that are caused by tDCS could also induce long-term changes in certain neuronal functions, leading to unintended effects which could be difficult to reverse (Cabrera et al., 2014; Fitz and Reiner, 2014).

### Assumptions About Efficacy and Actual Benefits

As previously discussed, tDCS for memory enhancement shows promising effects on the memory of healthy people. The conclusion that tDCS improves cognition in laboratory settings still does not mean that a transition to real life use is warranted. Potential benefits are thus debatable, as study designs on healthy individuals present some important limitations regarding their ecological validity (De Ridder et al., 2014; McKendrick et al., 2015). The ecological validity of experimental tasks needs to be improved. Namely, it is necessary to increase the control of external factors that could have an impact on expected effects (for example, acting under stress; Levasseur-Moreau et al., 2013). In addition, it should be noted that cognitive enhancement research on healthy participants has limitations: any enhancement effect may depend on the context, motivation, mental effort and individual differences (Schutter, 2014). These limits are also methodological: participants are volunteers, which has an impact on the expected effect (CCNE, 2014) and this restricts the transition from research to practice (Schutter, 2014). Moreover, it is questionable whether these technologies are powerful enough to have an actual cognitive impact in real-life settings, especially concerning their magnitude and duration (Levasseur-Moreau et al., 2013). Thus, it seems particularly appropriate to question the advertising of benefits of tDCS devices that are commercially available. For example, a recent study has shown that the use of one of these commercially available devices does not improve working memory as advertised, but in fact impairs this cognitive function in healthy study participants (Steenbergen et al., 2015). Thus, at least some of the benefits promised by producers of commercial tDCS devices have not materialized and may not be evidence-based. Finally, it will be necessary to compare the real benefits of tDCS to the efficacy of traditional memory enhancers (e.g., training, or sleep) to evaluate the actual contribution and advantage of this device (as proposed in Dresler et al., 2013).

### Assumption About Potential for Misuse of tDCS

Safety concerns are related to the assumption that private users of tDCS will misuse the device. Indeed, we could question the users’ expertise and worry about their capacity to choose the appropriate frequency, intensity and location of electrical stimulation. A recent study conducted by Jwa (2015) shows that tDCS users are actually aware of basic safety rules and are concerned about potential risks—especially risks of potential impairment of the brain and the use of incorrect positions of electrodes (Jwa, 2015). Furthermore, users gather information about risks and appropriate use in scientific literature. At the same time, users acknowledge the need for official or expert guidelines—even if they are concerned about excessive government regulation (Jwa, 2015). However, there are important limitations that should be mentioned. The single study on tDCS users relied on a convenience sample, which might bias the results. Also, the anonymous participants were mostly adults so the study might not adequately represent the perceptions of adolescents, who are more at risk for impulsive decision-making and misuse in different settings (Powell, 2006). While these limitations should be addressed in further studies, this study suggests that fully competent adult users of tDCS will most likely not misuse tDCS, as long as official guidelines and information about safe use is made available.

### Assumption About Clear and Complete Information About Risks

Various aspects discussed so far lead to a conclusion that providing clear and complete information on the risks of enhancement uses of tDCS is difficult, if not impossible, at this time. A first concern arises from the designation given to the technology: lack of knowledge about potential risks (as presented above) is particularly problematic since technologies such as tDCS are widely considered and described as “non-invasive” brain stimulation (Davis and van Koningsbruggen, 2013; Duecker et al., 2014). The notion of non-invasiveness could lead to misinterpretations that may confer a false sense of safety (Davis and van Koningsbruggen, 2013; Cabrera et al., 2014; Hildt, 2014). In presenting these devices as non-invasive, patients, participants or private users might overestimate benefits and underestimate risks (Davis and van Koningsbruggen, 2013; Hildt, 2014). On the other hand, recategorizing tDCS explicitly as “invasive” could be an exaggerated reaction, potentially inducing unfounded fear (Davis and van Koningsbruggen, 2013). It has been proposed that these devices be described as “brain stimulation techniques”, without mention of invasiveness (Davis and van Koningsbruggen, 2013). tDCS could also be described as “minimally invasive” (Cabrera et al., 2014), highlighting the fact that this technique imposes an exogenous current to the brain.

In addition to the described invasiveness of tDCS, other elements must be considered to ensure clear and accurate information. For example, risk-taking for enhancement is perhaps less justified given the lack of clarity regarding the real benefits for healthy users (Farah et al., 2004; Chatterjee, 2006; see “efficacy” above). The minimum level of acceptable risk in the case of cognitive enhancement is to be established for both daily applications and in research protocols involving healthy subjects: such an acceptable minimum should be reduced proportionately to the decrease in benefits for participants (Farah et al., 2004). Thus, given the lack of knowledge about risks and benefits associated with enhancement use of tDCS, some authors describe the behavior of current users as “self-experimentation” (Cabrera et al., 2014; Davis, 2016). Even in the case in which it is assumed that people using tDCS at home have made a deliberate choice to take the risk for themselves, it is currently impossible to make a truly informed choice, i.e., a choice based on reliable information about risks and benefits and about the parameters of appropriate stimulation (Hildt, 2014). Thus, the assumption that real and complete information will not be available for users is confirmed.

### Assumption About Prevalence of Use

Prevalence of tDCS enhancement research is something growing exponentially. Research has shown that 250 neuroscience studies using tDCS were published in 2013, compared to less than 50 in 2006 (Dubljević et al., 2014). Even if the high volume of scientific studies might suggest high prevalence of lifestyle uses, the number of private users of tDCS is still uncertain. We may suppose that there are thousands of one-time users and perhaps several hundred continuous users based on online availability and forum discussions (Jwa, 2015). For example, the company foc.us was out of stock due to high demand within a month: 3000 tDCS units were sold in the month of May 2013 (Jwa, 2015). However, this could not be considered as valid data for evaluating the actual prevalence of private tDCS use, as acknowledged by Farah (2015). As tDCS devices in various configurations are already available online and can be easily created with the help of readily available electronic components and YouTube tutorials, there is likely a small but growing population of at-home users. Given that enhancement with tDCS is not approved in clinical settings, the prevalence of clinical use is likely very low. An explicit requirement of use in specific contexts, such as educational, army or workplace settings, does not exist to date. The Defense Advanced Research Projects Agency’s (DARPA) growing interest in enhancement projects for neurotechnologies (such as tDCS), their large budget ($2.87 billion enacted budget for the year 2016) and the amounts they involve for academic research (estimated at around 90% of DARPA’s budget) suggests that the prospective concern about requirements of use might have some merit, at least in military contexts (see Moreno, 2012; Defense Advanced Research Projects Agency, 2016).

### Assumption About Social Acceptance

Social acceptance of tDCS is essentially reliant on a favorable public attitude toward performance improvement and memory enhancement uses. However, public attitudes toward enhancement have not been sufficiently investigated. One of the few studies on this topic has shown that the public, far from being ignorant and incompetent on such concerns, adopts a “biopolitically moderate” stance on the challenges related to the development of neuroenhancement (Fitz et al., 2013). Study participants’ reactions to the four main questions raised by neuroethicists were tested, i.e., regarding safety, fairness, authenticity and societal pressure (Fitz et al., 2013). More studies on this topic could lead to an understanding of a general opinion regarding this phenomenon, which may be extremely variable from one population to another, even within Western societies themselves. Public opinion was also investigated in a literature review (Dijkstra and Schuijff, 2016) and it was found that while there are debates in many countries (within Europe, North America and Asia) about public attitudes to human enhancement (see Jotterand and Dubljević, 2016), only 38 studies have been carried out over 13 years. This review showed that the public is moderately to strongly against non-medical uses of technology, that reasons which lead to these uses are perceived as worse when driven by personal goals than by social reasons, and that this attitude depends on the type of technology in question (Dijkstra and Schuijff, 2016). However, the above mentioned study by Fitz et al. (2013) addressed ethical concerns in a more comprehensive way. This study suggests that individuals have more propensity to accept risk when the goal is treatment than when it is to enhance performance without substantial differences in opinions between technologies (e.g., tDCS vs. pharmacological drugs; Fitz et al., 2013). In the same study, it was reported that “soft” peer pressure seems more problematic than pressure from society as a whole. Furthermore, authenticity is viewed in a consequentialist manner: merit is greater when observing a success, regardless of the means of getting there, but perception of worthiness is increased when performance is derived from hard work (Fitz et al., 2013). Differences in sampling and characteristics of populations investigated (online vs. traditional surveys, Europe vs. North America) might contribute to the diversity in results generated so far. More empirical studies of prevalence and public perception, and conceptual analyses of social and cultural norms across the globe are needed before firm conclusions can be drawn (Jotterand and Dubljević, 2016).

### Assumption About Ideological Stance

Ideological stances affect several aspects relevant for ethical reflection on tDCS. Most notably, in the absence of clear formal rules, the acceptance of informal rules which would designate the use of tDCS as cheating, or as legitimate, is crucially dependent on the ideological stance. The bioconservative stance highlights the potential negative impact cognitive enhancement can have on human achievement (Racine, 2010). Always extending the boundaries of the norm might lead to an endless quest in which no one is benefitting and everyone has to endure side-effects. At the same time, a biolibertarian approach considers such use as beneficent, and identifies many problems that these technologies could now solve such as performance anxiety, lack of sleep, and loss of attention. Also, for some authors, enhancement and virtue may coexist (Caplan, 2004). While it is indeed satisfying to overcome challenges pushing one’s own boundaries, it can also be satisfying to get profits that “fall from the sky” (Caplan, 2004). In other words, we do not always need to earn our happiness to be really happy, and the perceived authenticity of our actions is not necessarily a result of the suffering that went into their fulfillment (Caplan, 2004). However such claims could be counter to evidence gathered in social psychology where the intrinsic nature of motivations is associated with positive contributions to happiness and genuine self-fulfillment (Ryan and Deci, 2000). Furthermore, according to the biolibertarian approach, an individual must be free to choose to enhance their performance because there are allegedly no substantial differences with other types of improvement such as tutoring or exercise (Racine, 2010). So far, the actual improvement of well-being and happiness achieved by enhancement is questionable (Dresler et al., 2013; Madan, 2014; Nagel, 2014; Schleim, 2014). Also, there is some evidence to suggest that the public is neither strictly neoconservative nor bio-libertarian, but instead “bio-politically moderate” (Fitz et al., 2013), which puts the questions of which informal rules are actually accepted to the forefront of missing data that should be populated by empirical results.

### Most Plausible and Imminent Issues

In sum, most issues and sub-issues identified (see Table 2) are supported by modestly evidenced assumptions. Therefore, our analysis of underlying assumptions suggest that at least two issues/sub-issues, safety and autonomy (informed consent), are plausible and imminent. Indeed, safety concerns are not certain, but in view of the potential dangerous nature of unknown and unexpected side effects (there is no data to invalidate the ethical assumption), such concerns deserve to be duly considered in regulatory approaches. Additionally, we conclude that the worries about breach of informed consent are real and actual: there is not enough reliable information available for users and, in fact, users are perhaps misled by commercial enterprises selling tDCS devices.

**Table 2:** **Assessment of ethical issues of non-medical use of tDCS for regulatory purposes**.

High priority issues	Moderate-priority issues	Low priority issues
− Safety − Autonomy (informed consent)	− Autonomy (implicit coercion) − Distributive justice − Fairness	− Autonomy (explicit coercion) − Authenticity (individual) − Authenticity (collective)

Three assumptions, autonomy (implicit coercion), distributive justice, and fairness are less plausible and imminent, as the limited currently available data is insufficient to infirm or confirm the assumptions on which there are based.

Finally, three assumptions are much less plausible and are rather distant: autonomy (explicit coercion), authenticity (individual), and authenticity (collective). Indeed, explicit coercion is less urgent to address, as there is no current explicit requirement of tDCS in any specific context to date (here, actual data invalidate the assumption). Both authenticity concerns are highly prospective as currently available scientific knowledge is not sufficient to confirm assumptions about consequent efficacy and prevalence, and because such concerns are extremely dependent on ideological stance.

## Methodological Guidepost 3: Adopt a Broad Perspective to Support More Comprehensive Reflection

To adopt a broad perspective, Racine et al. (2014) suggest three relevant strategies to support a more reflective practice of bioethics: comparing disciplinary frameworks, considering historical knowledge, and reflecting on the development of normative approaches (Racine et al., 2014).

### Compare Disciplinary Frameworks

As acknowledged by Racine et al. (2014), one of the major concerns is that the terminology of “cognitive enhancement” reflects a specific dominant framework in bioethics which already implies some benefits on which we have very limited evidence. This dominant framework sometimes contrasts with other perspectives on non-medical uses of stimulants (e.g., within the public health literature, which emphasizes issues such as addiction or public harm). To analyze cognitive enhancement uses solely though the bioethics perspective—that could be extended to bioethics perspective of tDCS non-medical uses—may limit discussion and possible solutions. For example, concerns about distributive justice and fairness have to be addressed in regulatory approaches to tDCS according to future evidence on its efficacy for cognitive enhancement (in terms of memory and overall performance). This is because currently available scientific data does not confirm these assumptions. Therefore, such issues are thus highly dependent on the buy-in to the “cognitive enhancement” framework.

### Consider Historical Knowledge

As described by Racine et al. (2014), enhancement is not a new phenomenon. A historical perspective could help to assess social and cultural context of cognitive enhancement (Racine et al., 2014) with tDCS. Indeed, the trajectory of use of any enhancers seems to follow an enthusiasm/disillusionment cycle (Bell et al., 2012). This is exemplified by the history of pharmacological stimulants, such as cocaine and amphetamine: they first met an uncritical enthusiasm in view of the drug effects, until evidence of addiction and dangerous side effects emerged, which led to a sharp increase in societal concerns (Bell et al., 2012). Similarly, many innovations in electrotherapy (e.g., the “Electreat”, a transcutaneous electrical nerve stimulation device using electrical shocks in the early 20th century) have been the object of strong enthusiasm before the emergence of skepticism. Such skepticism surrounding the lack of scientific evidence of effectiveness and some use by opportunistic individuals taking advantage of public hopes (Basford, 2001). Thus, electric and magnetic approaches to treating disease and enhancing performance seem to follow the same kind of cycle, as noted by Basford (2001): “a pattern seems to reappear. In each era, unsophisticated public acceptance is met first with medical disdain, then with investigation, and, finally, with a failure to find objective evidence of efficacy” (Basford, 2001, p.1). At the same time, we should consider that every memory enhancement tool has been viewed as ethically problematic in a given period (Madan, 2014).

Moreover, considering the changing nature of standards, medical values, and goals in our contemporary societies, a strict line between what is “normal” and “pathological” is hardly possible (Maslen et al., 2014a; Pustovrh, 2014) and historical examples of the evolution of boundaries between enhancement and medical uses support some caution about the usefulness of this dichotomy. Specific diagnostic criteria define when a person suffers from a mental disorder, as established by the different editions of the Diagnostic and Statistical Manual of Mental Disorders (DSM-5, now in its fifth version). However, such criteria regularly change, and even today consensus is lacking on their definition. For example, homosexuality was defined as a mental disorder by DSM-II in 1968, and was given the rank of first sexual deviation (Minard, 2009). The establishment of new criteria for mental disorders in the latest DSM also caused reaction based on the concern that, according to the new criteria, everyone could be diagnosed with a mental disorder (Pearce, 2014). Considering the current phenomenon of a biomedicalization of society—and of under-performance in particular—(Le Dévédec and Guis, 2013), it is particularly difficult to restrict the risks and ethical issues of cognitive enhancement to individuals considered as “healthy”. This notion might ultimately refer to an overly broad (or limited) spectrum of individuals, and the blurred line between “normal” and “pathological” could make the implementation of regulatory policies on enhancers such as tDCS difficult.

Thus, what we learn from the history of science is that we should cautiously consider issues based on ideological stance and related to public acceptance for the purposes of normative evaluation. Indeed, ideological stances and public acceptance can move and change according to context and time. This leads us to acknowledge the need for a context-dependent approach in the implementation of policies with regard to issues such as authenticity. That said, historical knowledge should be carefully considered in ethical analysis. If previous conclusions about electrotherapy or memory enhancement gave a distorted picture, current expert opinion should be taken with a dose of skepticism, as previous and actual risks may not have been considered in the same way.

### Reflect on the Development of Normative Approaches

Finally, given the calls to provide regulatory responses to non-medical uses of tDCS, it is important to critically review policy proposals as well as their underlying normative assumptions, as recommended by Racine et al. (2014).

Maslen et al. (2014a) suggest extending the biomedical model to the enhancement uses of neurostimulation devices. This regulatory approach could be relevant because devices are the same for clinical use and for enhancement use—this is true for psychostimulants or DBS but less obvious for tDCS, which is not yet officially approved as a therapeutic tool—and because of the blurred and shifting line between the “normal” and the “pathological” (Maslen et al., 2014a). At the same time, by removing this distinction, the biomedical model reinforces the risk of distorting health priorities (CCNE, 2014). This risk is especially worrisome in a context where health resources are limited, raising the question of the legitimacy of using these resources in an enhancement context, namely for improving performance (De Ridder et al., 2014), especially since benefits and risks are uncertain (Forlini and Racine, 2012). Moreover, expanding biomedical approval to tDCS devices could create a false perception that these technologies are beneficial (De Ridder et al., 2014). In addition, such regulation does not include all modalities of tDCS. Unlike other biomedical technologies such as psychostimulants, tDCS is indefinitely reusable. The biomedical model should not be applied to tDCS only as a medical service, because this overlooks the fact that it also exists as a product and do-it-yourself gadget (Dubljević, 2015).

Another proposal from Fitz and Reiner (2013) is the creation of an official online forum where the community of tDCS users could receive advice and safety guidelines, with the ability to consult an expert scientist or clinician. This approach is guided by a principle of harm reduction, and aligns with current tDCS users’ requests for advice and expertise, as observed in unofficial online forums (Fitz and Reiner, 2014). It would have the advantage of being less intrusive in terms of state involvement and costs associated with its implementation in comparison to the first option described. However, this solution is not without costs, particularly related to the availability of experts (Fitz and Reiner, 2013). Since no additional specific measures are developed, the proposal implies the voluntary participation of health professionals. Managing risks through an online forum could also increase the number of tDCS users (Fitz and Reiner, 2014).

Some authors offer to frame tDCS devices for their personal use, without considering them as medical devices, by promoting awareness and public education (De Ridder et al., 2014), or even establishing a licensing procedure (Dubljević, 2015). To prevent the risks associated with misaligned stimulation intensity or positioning of electrodes, user manuals and training could be in place to ensure a minimum level of professional control before allowing private use, which could for example restrict the personal use of tDCS for trained users over 25 years (De Ridder et al., 2014; Dubljević, 2015). This kind of regulation would seek to limit doses and ages of users (De Ridder et al., 2014; Dubljević, 2015).

Different societies are likely to opt for different policies, and it may turn out that none of the proposals mentioned here are actually implemented. Indeed, our analysis should not be construed as arguing for a specific normative approach. However, the presence of plausible and imminent risks highlights the need for a broad discussion regarding development and implementation of policies, which would provide official guidelines and adequate information or education to users. At a minimum, such guidelines should present risks of tDCS use and real benefits to users, based on the actual level of knowledge supporting them. The lack of clarity on long-term side effects and efficacy of the devices in real life setting should also be conveyed by manufacturers and others involved in their commercialization.

## Conclusion

tDCS is a neurotechnology under intense investigation. From a rather marginal and obscure neurostimulation device, it has become the center of debates about the use of non-invasive neurostimulation for cognitive enhancement purposes. A number of important ethical issues are associated with cognitive enhancement uses of tDCS. Relying on a reflective pro-active methodology for bioethical analysis, we found that a number of assumptions presupposed by these issues are rather lacking in supporting evidence and in need of much greater scrutiny. For example, breach of informed consent (an autonomy-related issue) is one of the most imminent and actual ethical issues as we do not have enough data to ensure the availability of clear and complete information about risks and benefits. Meanwhile, authenticity issues are highly speculative and less urgent to address in terms of policy, because they are based on assumptions of high prevalence and consistent efficacy that are not supported by strong evidence. However, our analysis does not negate the importance of more speculative issues: even if such concerns are less urgent, they also deserve some attention and caution. Furthermore, we acknowledge that speculation has some important and relevant functions in neuroethics analysis, according to preventive approaches and proactive management of risks. We observed that applying different methodological strategies such as the comparison of disciplinary frameworks revealed that the discourse in which current discussions are embedded (e.g., “cognitive enhancement”) carries with it a number of assumptions that should be critically assessed as policy and regulatory responses to tDCS start to be debated and envisioned. Thus, we highlight the need for managing private use of tDCS without further delay and argue that relevant implementation of policies and regulation need first and foremost to take into account most urgent and realistic issues.

## Author Contributions

NV, VD and ER conceived the article together. NV and VD wrote the first draft, NV, VD and ER reviewed several versions and approved the final manuscript for submission.

## Conflict of Interest Statement

This work was partially funded by the Banting Postdoctoral Fellowships Programme (VD) and a grant from the Canadian Institutes of Health Research (ER, co-PI). Neither organization influenced the content of the manuscript in any way, and the manuscript should not be understood as representing the position of the funding bodies.

## Footnotes

^1^However, we should note that Western societies are not necessarily the most unequal, and that societies with large health and wealth inequalities could also have lower worker’s rights standards making this issue really international (Racine and Forlini, 2009).
